# Bridging technology and ecology: enhancing applicability of deep learning and UAV-based flower recognition

**DOI:** 10.3389/fpls.2025.1498913

**Published:** 2025-03-18

**Authors:** Marie Schnalke, Jonas Funk, Andreas Wagner

**Affiliations:** ^1^ Faculty of Management Science and Engineering, Karlsruhe University of Applied Sciences (HKA), Karlsruhe, Germany; ^2^ Fraunhofer Institute for Industrial Mathematics (ITWM), Kaiserslautern, Germany

**Keywords:** flower detection, deep learning, unmanned aerial vehicle (UAV), biodiversity, remote sensing

## Abstract

The decline of insect biomass, including pollinators, represents a significant ecological challenge, impacting both biodiversity and ecosystems. Effective monitoring of pollinator habitats, especially floral resources, is essential for addressing this issue. This study connects drone and deep learning technologies to their practical application in ecological research. It focuses on simplifying the application of these technologies. Updating an object detection toolbox to TensorFlow (TF) 2 enhanced performance and ensured compatibility with newer software packages, facilitating access to multiple object recognition models - Faster Region-based Convolutional Neural Network (Faster R-CNN), Single-Shot-Detector (SSD), and EfficientDet. The three object detection models were tested on two datasets of UAV images of flower-rich grasslands, to evaluate their application potential in practice. A practical guide for biologists to apply flower recognition to Unmanned Aerial Vehicle (UAV) imagery is also provided. The results showed that Faster RCNN had the best overall performance with a precision of 89.9% and a recall of 89%, followed by EfficientDet, which excelled in recall but at a lower precision. Notably, EfficientDet demonstrated the lowest model complexity, making it a suitable choice for applications requiring a balance between efficiency and detection performance. Challenges remain, such as detecting flowers in dense vegetation and accounting for environmental variability.

## 1 Introduction

The decline of insect biomass, including pollinators, by more than 75% in 27 years (Hallmann et al., 2017) represents a significant ecological challenge with long-term implications. Research has shown that this decline is negatively impacting plant populations, further highlighting the vital role of pollinators in maintaining ecosystem stability (Kevan and Viana, 2003; Thomann et al., 2013; Ramos-Jiliberto et al., 2020). These findings emphasize the urgency of continued research and consistent monitoring of both pollinator and plant populations. This study contributes by enhancing the applicability for monitoring floral resources in grassland ecosystems using deep learning models and drone technology. Floral resources and their diversity are pivotal to the composition and abundance of bee communities (Potts et al., 2003). A diverse floral landscape not only reduces competition among pollinators, but also accommodates the unique foraging preferences of different species, promoting overall ecosystem health (Bergamo et al., 2020). For example, Torné-Noguera et al. (2014) found that certain bee species have preferences for specific flower types, emphasizing the importance of floral diversity for understanding pollinator distribution. Similarly, Torresani et al. (2024) demonstrated a positive correlation between increased vegetation height heterogeneity and higher species diversity in both flowers and pollinators, using drone imagery. In recent years, the use of drones, technically referred to as UAVs, to monitor floral resources has been increasingly explored. A notable example is the work of Anderson et al. (2024), who used UAVs to quantify flower coverage by analyzing aerial images. This method proved effective in accurately determining the percentage of area covered by flowers in large landscapes, highlighting the growing importance of drone technology in large-scale floral resource monitoring. While UAVs and other remote sensing technologies have been widely used in agricultural research to monitor plant health, yield, and biodiversity over large areas (Lyu et al., 2022), their application in pollination ecology remains limited (Willcox et al., 2018).

The *BeeVision* project^1^ deals with the decline of pollinators and develops innovative, non-invasive approaches for monitoring biodiversity. One central idea of this project is to integrate floral resources as a variable within geostatistical methods to improve pollinator abundance interpolation. This approach is similar to the work of Monfared et al. (2013), where additional variables such as altitude and temperature were incorporated to improve prediction accuracy. The geostatistical application requires an accurate and non-invasive measurement of floral resources over a large area.

Traditionally, environmental data collection, including the counting of flowers, is done manually. However, this approach becomes increasingly difficult and inefficient when applied to large landscapes, as it is both time consuming and resource intensive (Pettorelli, 2013). As a result, recent developments have focused on automatic flower detection methods. Such advances are critical to improve conventional methods of identifying biological objects. Automating the identification of plants and flowers is a promising approach to reduce reliance on human experts and increase accuracy (MacLeod et al., 2010), especially in combination with drones. Many studies focus on counting a specific type of flower. For example, Xu et al. (2018) recognized and counted cotton flowers, while Liang et al. (2018) and Wan et al. (2018) focused on rapeseed flowers. Petrich et al. (2020) proposed a method to detect and localize *Colchicum autumnale* using drone images. Localization is particularly useful for the development of targeted measures in agricultural plant monitoring. Other studies extend the focus beyond a single class to include classification of multiple flower classes. For example, Sarkar and Kelley (2023) presented an approach to distinguish between native and invasive plant species by analyzing 20 native and 18 invasive classes on RGB images captured with a DJI Air 2S. Gallmann et al. (2022) also classified different classes of flowers and developed a toolbox^2^, hereafter referred to as the Gallmann Phenotator Toolbox, for automatic detection and classification of flowers using a Faster R-CNN (Ren et al., 2016) that was trained on drone images of flowers in grasslands. This tool is based on a detection model from the Detection Model Zoo^3^ of the TF^4^ Object Detection API^5^. The corresponding code is well documented to facilitate annotation and data preparation of images, as well as training, testing, and evaluation of models. With their Faster R-CNN, they achieved an overall accuracy of 87% and a recall of 84.2% on test data. In addition, the study mentioned the applicability of the tool’s predictions to a larger grassland area by creating orthomosaics. However, the software packages and dependencies used in this tool are now obsolete^6^.

The work of Gallmann et al. (2022) was chosen as a foundation due to its strong emphasis on usability. Practical applicability is a priority for the goals of this study, and the use of pre-trained models simplifies the workflow for biologists, allowing them to make predictions directly from drone imagery once the Gallmann Phenotator Toolbox is established. In addition, the study considered different flower classes, which, as highlighted above, are essential for maintaining pollinator diversity. Given the outlined importance of monitoring plants and pollinators, this research provides the following contributions:

1. Toolchain Update: We updated the GitHub Code of the Gallmann Phenotator Toolbox, integrating recent software packages to ensure compatibility and practical use. Additionally, we extended the toolbox by incorporating two additional models from the TF2 Model Detection Zoo, namely EfficientDet (Tan et al., 2020) and SSD (Liu et al., 2016), to further enhance compatibility with further models.
2. Comparative Analysis of Models: We performed a comparative analysis of three different object detection models from the TF 2 Model Detection Zoo (EfficientDet, Faster R-CNN and SSD) presenting differences in detection performance.
3. Practical Guidelines for Biologists: We developed practical guidelines for biologists to facilitate flower detection in grasslands, bridging the gap between machine learning and fieldwork.

With these contributions, this study aims to improve the applicability of UAV-based methods for automatic flower detection in species-rich grasslands. In order to achieve these goals, this paper is structured as follows. Section 2 outlines the methodology, starting with a literature review on flower recognition and classification, followed by a discussion on the use and limitations of UAVs in remote sensing. This section also covers the training and testing of the three object recognition models, and the development of practical, field-ready guidelines for biologists. Section 3 presents the results of the model comparison, highlighting the most efficient model. Section 4 discusses the results in terms of the strengths and weaknesses of the models, highlights challenges, and suggests future extensions. Section 5 concludes the main findings.

## 2 Materials and methods

### 2.1 Literature review

To contextualize and understand the progress and challenges in UAV-based flower detection and classification, it is important to examine how flower detection methods have evolved over time. Initially, these methods were based on classic computer vision techniques that focused on analyzing and extracting image features such as color and texture using simple mathematical approaches. For example, Adamsen et al. (2000) developed a fully automated system for non-destructive flower counting using digital camera images and the Euler method, which resulted in a time savings of 92% per image compared to manual counting. Also threshold analysis emerged as an effective technique for separating peach (Horton et al., 2017) or canola (Zhang et al., 2021) flowers from the background. In a different approach, Hsu et al. (2011) developed an interactive flower recognition system that allows the user to define an area of interest in which the flower is located. The area is segmented and the extracted features - such as color and shape - are statistically analyzed and compared to a database of flower images. The flower class is identified based on the smallest Euclidean distance between its features and those of the input image. As the field progressed, machine learning algorithms were introduced to further enhance the process of classification. For instance, Nilsback and Zisserman (2008) improved flower recognition by combining multiple feature extraction techniques with a support vector machine (SVM), leading to a significant boost in accuracy. They explored the importance of different features for distinguishing between several similar classes of flowers and achieved a 12.7% increase in accuracy by combining four distinct features^7^ compared to using a single one. Siraj et al. (2010) compared the performance of a logistic regression model for feature extraction with that of a neural network. The neural network outperformed the logistic regression, achieving 41.83% higher accuracy in classifying flower images. Building on the foundational work of early neural networks, recent advances in deep learning, particularly through the use of convolutional neural networks (CNNs) (O’Shea and Nash, 2015), have revolutionized image processing (Ye, 2024). Unlike traditional neural networks, CNNs are specifically designed to recognize and process spatial patterns in images. Their architecture of local connections and parameter partitioning not only increases efficiency, but also minimizes computational requirements (O’Shea and Nash, 2015). These models have significantly enhanced the capabilities of flower recognition, delivering very good results. For example, Xu et al. (2018) reported an accuracy of more than 97% when applied to cotton flowers and the best CNN model from Sarkar and Kelley (2023) achieved an accuracy of 94%. Further extending CNN capabilities, models like Faster R-CNN have been adapted for real-time applications in various environments. Patel (2023) employed this model with a ResNet50 backbone to accurately recognize the flowering stages of marigolds in real-time field scenarios. Additionally, Abbas et al. (2022) compared different backbones for Faster R-CNN against SSD for flower detection and classification using digital camera images. Their findings revealed that the Inception V2 backbone demonstrated the best performance with a mean Average Precision (mAP) of 91.3%. Beyond these applications, John et al. (2024) explored YOLO, RetinaNet, and Mask Region-Based Convolutional Neural Network (Mask R-CNN) to determine the biodiversity of mountain meadows. Furthermore, Basavegowda et al. (2024) used EfficientDet, trained on data from greenhouses and grasslands, to detect High Nature Value (HNV) indicator plants in semi-natural grasslands. This model was specifically trained with nadir perspective data to facilitate future research, including studies using UAVs.

The advances in flower detection and classification have greatly improved the accuracy of these methods. To apply them to larger and difficult-to-access areas, innovative monitoring and data collection approaches, such as UAVs, are necessary (Pettorelli, 2013; Wan et al., 2018). Capture devices for flower detection range from smartphones (Wu et al., 2020; Shang et al., 2023; John et al., 2024) to digital cameras on ground vehicles (Ozcan et al., 2020). For large-scale analysis, remote sensing technologies such as satellite imagery (Landmann et al., 2015), manned vehicles (Barnsley et al., 2022), and UAVs (Wan et al., 2018; Xu et al., 2018; Zhang et al., 2021) are becoming increasingly important. In particular, UAVs enable efficient and large-scale data collection that is faster (Xu et al., 2018) and less resource-intensive than traditional, error-prone methods (MacLeod et al., 2010; Pettorelli, 2013). Furthermore, UAVs offer a standardized setup for automatic data collection, allowing for repeated measurements at the same location with minimal effort (Duro et al., 2007). Through their physical distance from the ground, UAVs also provide a non-invasive way to collect data (Xu et al., 2018). In addition, they offer a cost-effective alternative for environmental monitoring (Huang et al., 2013; Anderson et al., 2024), helping to alleviate the high costs typically associated with automated flower detection systems - a burden typically shouldered by public institutions (Gaston and O’Neill, 2004). By expanding the capabilities of automated detection and classification applications, UAVs are now being applied to a broad range of targets. These include fruit detection (Chen et al., 2017; Bellocchio et al., 2019; Akiva et al., 2020; Zhao et al., 2024), tree flower detection and quantification (Carl et al., 2017; Horton et al., 2017; Zhang et al., 2023; Shang et al., 2023), as well as flower identification, monitoring, and counting in grasslands (Petrich et al., 2020; Gallmann et al., 2022). Xu et al. (2018) were the first to introduce flower counting using UAV imagery. Despite its advantages, using UAVs for flower counting presents challenges, particularly in detecting smaller or obscured flowers due to the lower resolution compared to ground-based imagery. Even with manual counting in drone imagery, these issues persist, although the risk of human error, such as overlooking or double-counting (MacLeod et al., 2010) flowers, is reduced (Gallmann et al., 2022).

Overall, the cited studies show that deep learning models, especially when combined with UAV technology, are powerful and reliable tools for efficient evaluation of flower resources. Given the growing adoption of these methods for flower detection and classification, the Gallmann Phenotator Toolbox has been updated and extended by integrating two additional deep learning models and performing a comparative analysis of their accuracy on drone imagery. The extension allows expanding compatibility of the Gallmann Phenotator Toolbox with a wider range of models of the TF model zoo.

### 2.2 Resources

**Figure 1:**
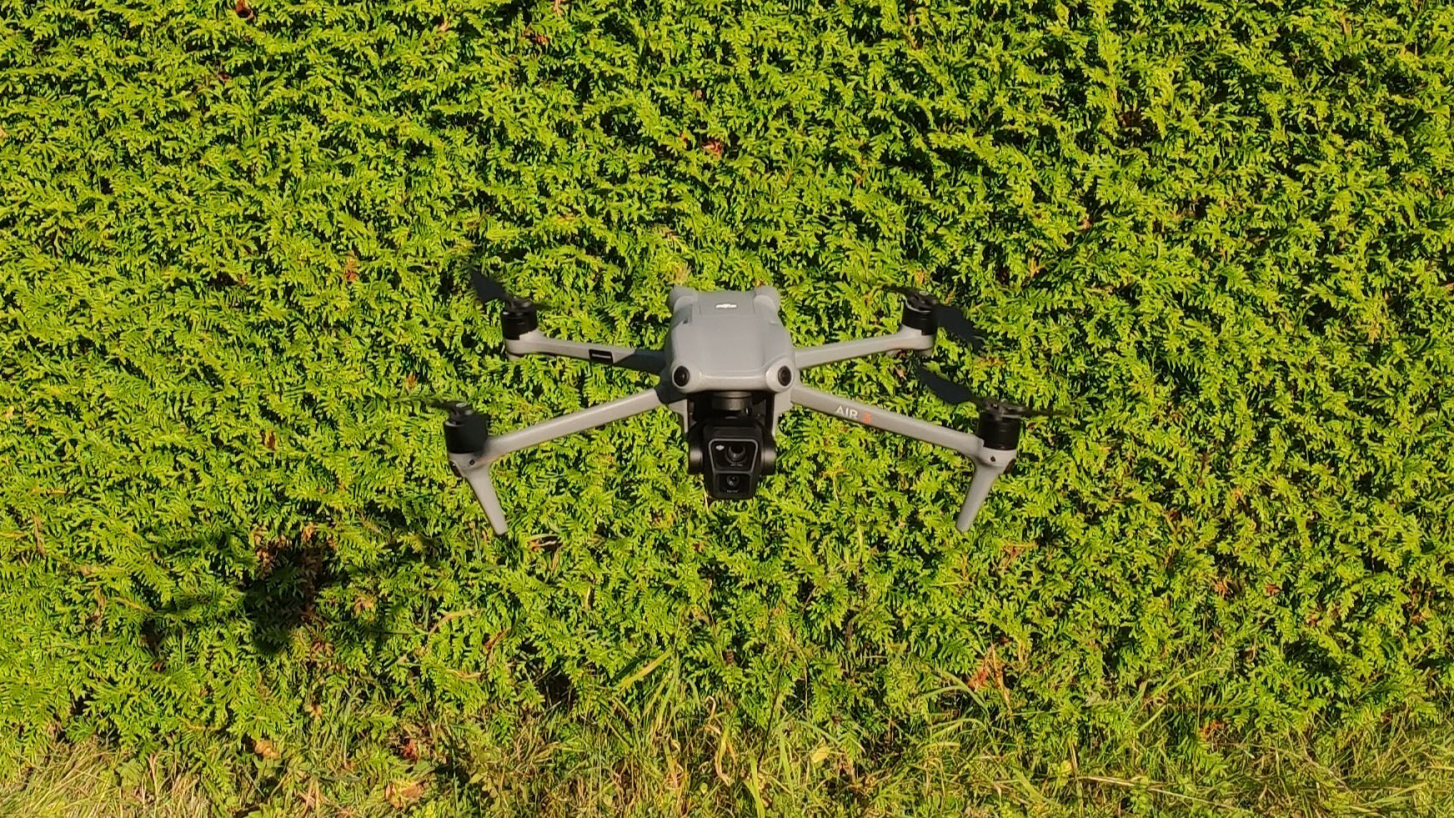
DJI Air 3: Drone used for capturing new aerial imagery for testing purposes.

Given the proven effectiveness of drones for large-scale, non-invasive data collection, a drone was used to collect additional test data. As mentioned above, UAVs are particularly advantageous in areas that are difficult or inaccessible to humans (Feng et al., 2021). The use of the drone in the test field proved to be extremely useful, as the tall grass poses a challenge for ground-based flower counting methods - treading on the vegetation flattens it, which can affect the accuracy of the survey. The drone allowed us to collect data without disturbing the natural state of the field. However, it was necessary to maintain a certain altitude, as the wind generated by the drone can cause movement in the grass, resulting in visual noise in the images (Stojnić et al., 2021). For this study, the DJI Air 3 (see **Figure 1**) was chosen as the primary tool for capturing images from the drone. This easily maneuverable rotary-wing UAV allows for efficient flower collection as it does not require a launch or landing site and can take off directly in the field. It also has an automatic return function. The drone can take high-resolution images while hovering over the area at low altitude. However, the short battery life, which requires frequent recharging, limits the efficiency and range of data collection (Shi et al., 2019). Under optimal conditions, the DJI Air 3 offers a maximum flight time of 46 minutes and a maximum flight distance of 32 km. Its internal memory is 8 GB and can be expanded with an SD card. A key reason for choosing this drone was its dual camera system, consisting of a 1/3-inch CMOS 48 MP wide-angle and a medium telephoto camera^8^. This combination makes it possible to capture both wide-angle and detailed close-up images of flowering areas for more accurate and efficient automated flower detection. UAVs with RGB sensors provide images that can quickly cover large areas and are easy to operate, making them a cost-effective method for monitoring grasslands (Sweet et al., 2022). At 720 grams, the DJI Air 3 qualifies for the “open” category under EU Regulation 2019/947^9^. This category is designed for low-risk operations, provided the pilot completes online training and passes a basic theory test. This minimal certification process makes drone technology widely accessible and encourages wider adoption for environmental monitoring.

While the UAV facilitated efficient data collection in the field, the training of the three models was performed using a machine learning hardware. It was equipped with two AMD EPYC 7742 central processing units (CPUs, 64 cores each), eight NVIDIA RTX A6000 graphics processing units (GPUs, 48 GB VRAM per GPU) connected via NVLink, and 768 GB of random access memory (RAM). As part of this work, the code base was updated to TF 2, following the official recommendations from the TF developers. Specifically, TF 2.15 was selected to ensure compatibility with CUDA 12.2 and cuDNN 8, as specified in the official TF table^10^. This combination of software versions was installed on the available server, allowing the integration and use of GPU resources for training and running the models^11^.

### 2.3 Updating the Gallmann Phenotator Toolbox

The Gallmann Phenotator Toolbox is based on TF 1, a powerful framework for machine learning. It was designed to enable scalable and efficient computations in heterogeneous environments by optimally allocating computational resources and managing the state of variables (Abadi et al., 2016). However, with the evolution of machine learning technologies, TF 2 has introduced several enhancements offering a more intuitive, adaptable, and robust framework. The improvements over its predecessor include the following^12^:

- Eager Execution: Eager execution, enabled by default, optimizes model development and debugging by allowing operations to be evaluated immediately, rather than building computational graphs^13^.
- Enhanced GPU Performance: TF 2 offers improved GPU performance, making it more compatible with modern hardware setups.
- Integration of Newer APIs: TF 2 introduces several major APIs. The Keras library is fully integrated as a high-level API^14^, providing a more intuitive interface that simplifies the development and training of deep learning models. The updated Object Detection API provides access to state-of-the-art models with various backbone architectures from the detection model zoo^15^. In addition, TF 2 offers improved performance for handling large datasets^16^ and optimizes the use of multiple GPUs.^17^

To take advantage of these new features and optimizations and to ensure compatibility with the latest software packages, the migration of the Gallmann Phenotator Toolbox code base to the newer TF version was required. TF 2 eases migration by providing comprehensive guides, documentation, and tools specifically designed to help developers transition from TF 1 to TF 2^18^. The migration involved identifying incompatible code sections, particularly deprecated functions and outdated APIs, and updating them to their TF 2 counterparts to ensure full compatibility with the new framework. In addition to updating the Gallmann Phenotator Toolbox with newer software, the code was extended to support not only Faster R-CNN, but also EfficientDet and SSD models. Their differences and advantages are further outlined in Section 2.6 (Model Training).

After successfully updating and extending the Gallmann Phenotator Toolbox for better and extended performance and compatibility with modern hardware, the source code was made available in a GitHub repository^19^. The next step in the application was to compile a comprehensive dataset. The datasets used serve as the basis for training and evaluation of the models. In the following section, the datasets are presented in detail to provide a basis for the following analyses.

### 2.4 Datasets

For this study, two datasets were utilized. To train and evaluate the models, the dataset from Gallmann et al. (2022) was selected. It consists of drone images of 1 m^2^ test squares captured from a height of 19 meters. A different drone and camera were used compared to the setup of this study. The dataset contains several flower classes (see **Table 1**), with some classes combined due to their visual similarity. It is thoroughly annotated with these flower classes.

**Table 1:** Flower species comparison: comparison of the presence of flower species in the Gallmann and Hohenheim Datasets.

Flower Species	Gallmann Dataset	Hohenheim Dataset
Agrimonia eupatoria	×	
Anthyllis vulneraria	⊗	
**Centaurea jacea**	⊗	×
Centaurea jacea	×	
Lychnis flos cuculi	×	
Cerastium caespitosum	×	
Cirsium arvense		×
Convolvulus arvensis		×
Crepis biennis	⊗	
Leontodon hispidus	×	
Picris hieracioides	×	
Tragopogon pratensis	×	
Dianthus carthusianorum	⊗	
**Galium mollugo**	⊗	×
Achillea millefolium	×	×
Carum carvi	×	
Daucus carota	×	
Galium mollugo	×	
Geranium dissectum		×
Geranium palustre		×
Geranium pratense		×
Knautia arvensis	⊗	
Leucanthemum vulgare	⊗	
**Lotus corniculatus**	⊗	×
Lathyrus pratensis	×	×
Lotus corniculatus	×	×
Medicago lupulina	×	
Onobrychis viciifolia	⊗	
Orchis species	×	
Plantago lanceolata	×	×
Plantago major	×	×
Prunella vulgaris	⊗	
Ranunculus	⊗	
Ranunculus acris	×	
Ranunculus bulbosus	×	
Ranunculus friesianus	×	
Rhinanthus alectorolophus	⊗	
Salvia pratensis	⊗	
Senecio spec.		×
Trifolium pratense	⊗	
Trifolium repens	×	
Veronica chamaedrys	×	
Vicia sativa	×	×
Vicia sepium	×	×

The circles indicate the classes on which the models were trained. In addition, the three classes that were included in the model training that are also present in the Hohenheim Dataset are highlighted in bold.

**Figure 2:**
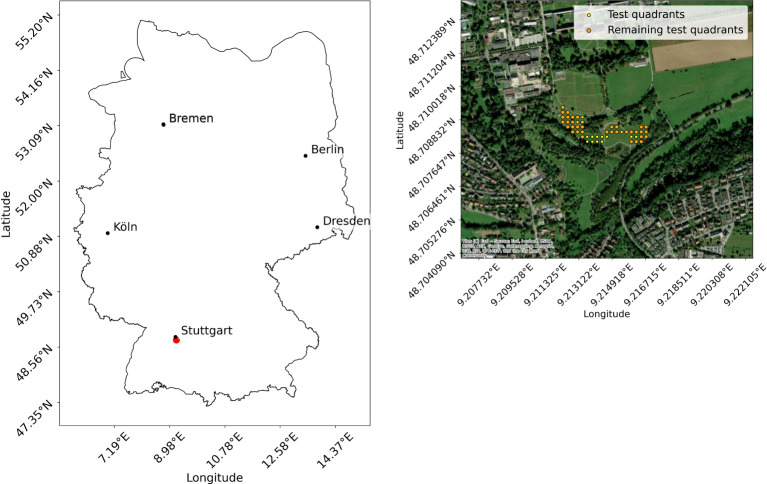
Location of data collection for the Hohenheim Dataset: The left image shows the location in Germany, and the right image shows the locations of the test quadrants. The map was created using geodata from OpenStreetMap^20^.

To obtain additional test data, the Hohenheim Dataset, an experiment was conducted. The data collection took place at the Hohenheim Gardens of the University of Hohenheim in Baden Wuerttemberg, Germany (coordinates: 48°42’29.6”N, 9°12’50.1”E) (see **Figure 2**) on July 16, 2024, between 13:35 p.m. and 16:05 p.m. The weather was sunny to cloudy with an average temperature of 31.6°C, an average global radiation of 800W/m^2^ and an average wind speed of 12 km/h, which is within the endurance range of the drone. The grass of the test site was tall and dry. The light conditions varied slightly due to intermittent cloud cover. Care was taken to ensure that the drone’s shadow is not visible within the test field. The test site had a biodiversity of 17 different flower classes, nine of which match the flower classes of the Gallmann Dataset (see **Table 1**). The experiment was designed as follows. In an area of 18.400 m^2^ (1.84 ha), 50 smaller plots of 1 m^2^ each were marked out. Due to the use of the wide angle camera for some images, where no flowers could be identified, only 36 plots remained for analysis (see **Figure 2**). Each plot was marked using wooden stakes to ensure minimal environmental impact. Ground truth data were collected manually by observers at each plot, where all flower species were identified and counted. This step allowed us to verify the accuracy of the drone data with field observations, using standardized data sheets to ensure consistency. In addition to the number of instances of each flower class counted, the number of insects flying to the flowers were counted. Results show that the collected flower classes are attractive to pollinators. Subsequently, drone images of each test plot were captured. To maximize detection accuracy, it is crucial that the image quality is high enough to clearly identify the flowers. This requires sufficient image resolution to clearly capture the fine features of the flowers (Xu et al., 2018). As described in Gallmann et al. (2022), the ground sampling distance (GSD) for flower recognition should be a maximum of 5 mm/pixel, as up to this value the prediction performance only changes slightly. Ideally, the images used for analysis should maintain a GSD similar to that of the training images to ensure consistent flower sizes. The images of the Gallmann Dataset, including training images, have a GSD of approximately 1.5 mm per pixel, while the images acquired for the Hohenheim Dataset have a GSD of approximately 1 mm per pixel. In addition to the images, videos of the test site were recorded with the goal of creating an orthomosaic. For this purpose, a predefined route was set in the included DJI Fly app. The drone flew over the site at the same altitude used for image acquisition, maintaining a speed of 1.9 m/s, as specified in the study by Li et al. (2023). The wide angle camera was used for video recording to cover as much of the area as possible. As a result, the resolution is lower, at 3840 x 2160 pixels. An orthomosaic was created using Web Open Drone Map (WebODM)^21^.

### 2.5 Image preprocessing

To prepare the images for model training, the Gallmann Dataset was deterministically divided into 70% training data, 10% validation data, and 20% test data. To ensure sufficient representation of each class in the training set, any class with fewer than 50 instances was excluded, following the approach of Gallmann et al. (2022). Additionally, in this study the image preprocessing script was modified to exclude any class with zero instances in the training set, ensuring that only classes present in the training data were used. This resulted in 14 classes left for training (see **Table 1**). Gallmann et al. (2022) originally resized the input images into 450 x 450 pixel tiles, which were then upscaled to 900 x 900 pixels. This method is consistent with the recommendations of Hu and Ramanan (2017), who advocates dividing images into smaller sections and upscaling them when detecting small objects. In contrast, this study applied a resizing strategy using larger dimensions. For example, Liu et al. (2016) emphasize the importance of input size for small object detection, showing that larger input sizes improve detection performance compared to smaller ones. Their experiments showed that increasing the input size from 300 x 300 pixels to 512 x 512 pixels improved mAP by 2.5%. Based on these results, an input size of 512 x 512 pixels was chosen. To further increase the resolution and detail of the images, especially for recognizing very small flowers, for this study, the input images were scaled to 1024 x 1024 pixels before training. This additional scaling aims to improve the detection of finer details.

### 2.6 Model training

All three models relevant for model comparison were trained on the annotated data of the Gallmann Dataset. The Faster R-CNN model from the TF 1 Model Zoo used a manual learning rate by default, which was adopted by Gallmann et al. (2022). However, a cosine decay learning rate of 0.08 was chosen for this work. This learning rate adapts automatically during the training process, allowing for dynamic adaptation. In general, the default configurations of the models were used, with a few adjustments made to improve performance. Specifically, the maximum number of detections per class and the total number of detections were both increased to 300, and gradient clipping by norm was applied with a value of 10.0 to stabilize training. The models were trained with a batch size of 4 for at least 200,000 steps^22^. Performance was evaluated every 2,500 steps. EfficientDet required an additional 50,000 steps due to the learning rate not converging earlier. The model that achieved the highest F1-Score was saved separately in a designated folder for further analysis. The results of the analysis are detailed further in Section 3 (Results). The TF 2 Detection Model Zoo is a comprehensive collection of pre-trained object detection models based on the Common Objects in Context (COCO) 2017 dataset (Lin et al., 2014). Each model is available with different backbones, making them versatile tools not only for inference on new data, but also as starting points for training on new datasets. The model zoo contains a variety of models, including the aforementioned Faster R-CNN, SSD, EfficientDet, CenterNet (Duan et al., 2019) and Mask R-CNN (He et al., 2018), each of which offers different trade-offs between accuracy and speed^23^. The first model analyzed is Faster R-CNN, which was also used in the study of Gallmann et al. (2022). The model is a two-stage detector that performs object detection in two steps. Its region proposal network (RPN) scans the input images of any size to generate region proposals, which are areas in the image where potential objects might be located. In a second step, the model classifies the objects within these proposed regions (Ren et al., 2016). The Faster R-CNN available in the TF 2 Model Zoo differs slightly from the TF 1 configuration used by Gallmann et al. (2022). The following data augmentation techniques were used in this study:

- Random horizontal and vertical flip
- Random brightness, contrast, hue and saturation adjustment
- Random crop and scale
- Random jitter boxes

Liu et al. (2016) emphasizes the importance of data augmentation in detecting small objects especially for SSD models. Compared to the Faster R-CNN, a SSD combines object localization and classification into a single step. Instead of using a region proposal network that adjusts anchor boxes to suggest possible object regions, the SSD model uses predefined default boxes with different aspect ratios and sizes that are placed on the feature map^24^. For each of these default boxes, the model computes probability scores for all object classes, indicating how likely it is that an object of a given class is located within the box. These default boxes are then refined by applying calculated offsets to generate the final bounding boxes that more accurately enclose the detected objects (Liu et al., 2016). SSD outperformed Faster R-CNN on the PASCAL VOC 2007 dataset (Everingham et al., 2010), delivering better accuracy and faster processing times (Liu et al., 2016). The third model in the comparison, EfficientDet, is also a single-stage detector designed for high efficiency and accuracy. Its key innovation, the Weighted Bi-directional Feature Pyramid Network (BiFPN), dynamically adjusts the importance of different features during training, allowing the model to focus on the most relevant information. By tightening connections and eliminating unnecessary paths, EfficientDet optimizes both computational power and accuracy. This makes it ideal for real-time analysis and resource-constrained applications (Tan et al., 2020).

Once the models are trained, the next step is to ensure their effective use in the field. Hodgson and Koh (2016) highlight the need for clear, actionable guidelines for UAV use in research. Building on the documentation in the Gallmann Phenotator Toolbox, step-by-step instructions are provided to help users accurately identify flower classes and numbers in meadows, ensuring ease of use in field studies.

### 2.7 Practical guidelines for UAV-based flower detection

In this section, the key steps for using UAVs in flower detection are outlined, including pre-flight setup, flight execution, and post-flight data processing. These guidelines ensure transparency, reproducibility and enable integration with machine learning models for flower classification in Germany. Before conducting drone operations, certain resources are required. These include a drone, such as the DJI Air 3, equipped with the necessary cameras, as well as a server for data storage, model training and evaluation. Additionally, operators must comply with the above mentioned EU Regulation 2019/947, which outlines requirements such as registration and certification for drone pilots. In addition, national regulations, including Germany’s Air Traffic Regulations (LuftVO)^25^ and the German Air Traffic Act (LuftVG)^26^, mandate requirements such as liability insurance. Drones in the ‘open’ category require operator registration and a pilot’s license. In addition, operators should check geographic restrictions such as no-fly zones or altitude restrictions^27^. Once all legal requirements have been met and a flight area has been selected, safety must be ensured by avoiding bystanders and strictly following regulations. During pre-flight preparations, camera settings must be configured to capture essential metadata, including GPS coordinates, altitude, speed, and timestamps for each image or video frame, the subtitle feature should be enabled in the drone’s camera settings, generating an SRT file during video recording (DJI, 2023). This metadata is essential for subsequent georeferencing and accurate orthomosaic creation. To enhance the accuracy of the georeferencing process, the use of ground control points (GCPs) should be considered (Gallmann et al., 2022; Sweet et al., 2022; Zhang et al., 2023; Torresani et al., 2024). Flight height should be carefully determined based on the size of the objects to be detected, the acceptable wind generated by the UAV, and the area to be covered (Stojnić et al., 2021). Minimizing environmental disturbance is also critical to reduce the impact on wildlife and the surrounding ecosystem, as recommended by Hodgson and Koh (2016). For flower imaging with the DJI Air 3, a height of 15 meters has proven effective for capturing images. Since video footage has a lower resolution than still images, a lower altitude is recommended. To increase efficiency and conserve battery power, it is advisable to use waypoints. These can be planned before the flight, enabling the drone to automatically follow a predefined route, or set at specific positions during the flight (DJI, 2023). Other tools, such as DroneDeploy^28^, offer waypoint planning with integrated orthomosaic generation. Finally, automatic settings for altitude, gimbal angle, and speed should be used to ensure consistent image quality. These parameters can be configured in the DJI Fly app before the flight, with the gimbal set to - 90° to provide a vertical view of the terrain. After completing pre-flight preparations, the drone will autonomously follow the predetermined flight path. It is important to make sure that the battery is fully charged and the weather conditions are stable before taking off (DJI, 2023). During flight, the drone’s status is monitored by the controller, allowing intervention if unexpected situations arise. Once the flight is complete, the captured data - including images, video, and metadata - should be transferred from the SD card to secure storage. It is recommended to back up the data in multiple locations to avoid potential loss. While the creation of an orthomosaic is optional, it can greatly assist in post-flight analysis for large areas. Tools like WebODM, a free and open-source solution, offer reliable alternatives to expensive commercial software for photogrammetry processing (Vacca, 2020). Creating orthomosaics is a standard practice in many UAV-based studies, with several researchers using commercial software solutions such as Agisoft Metashape (Gallmann et al., 2022; Zhang et al., 2023; Torresani et al., 2024), Pix4D (Zhang et al., 2021; Anderson et al., 2024), and DroneDeploy (Horton et al., 2017). In contrast, this study uses WebODM, which offers photogrammetric processing capabilities comparable to the commercial options, providing reliable photogrammetric processing on low-cost drone imagery (Vacca, 2020). In order to identify flowers on the created orthomosaic, it may be necessary to divide the large image into smaller sections if the maximum number of 178,956,970 pixels to be processed by the Phenotator Toolbox TF2 is exceeded. For analysis, regions of interest (ROIs) can be selected using the Gallmann Phenotator Toolbox. If adequate resources are available, custom models can be trained; otherwise, pre-trained models can be used. Ideally, when custom models are trained, they should be tailored to the specific flower species present in the meadow being analyzed. Predictions can be made on the orthomosaic or on selected ROIs (Gallmann et al., 2022). The results can then be visualized in a dashboard, which is introduced as a new feature in the Phenotator Toolbox TF2, offering a clear overview of detected flower classes, facilitating interpretation of results and supporting decision making in ecological studies.

## 3 Results

**Figure 3:**
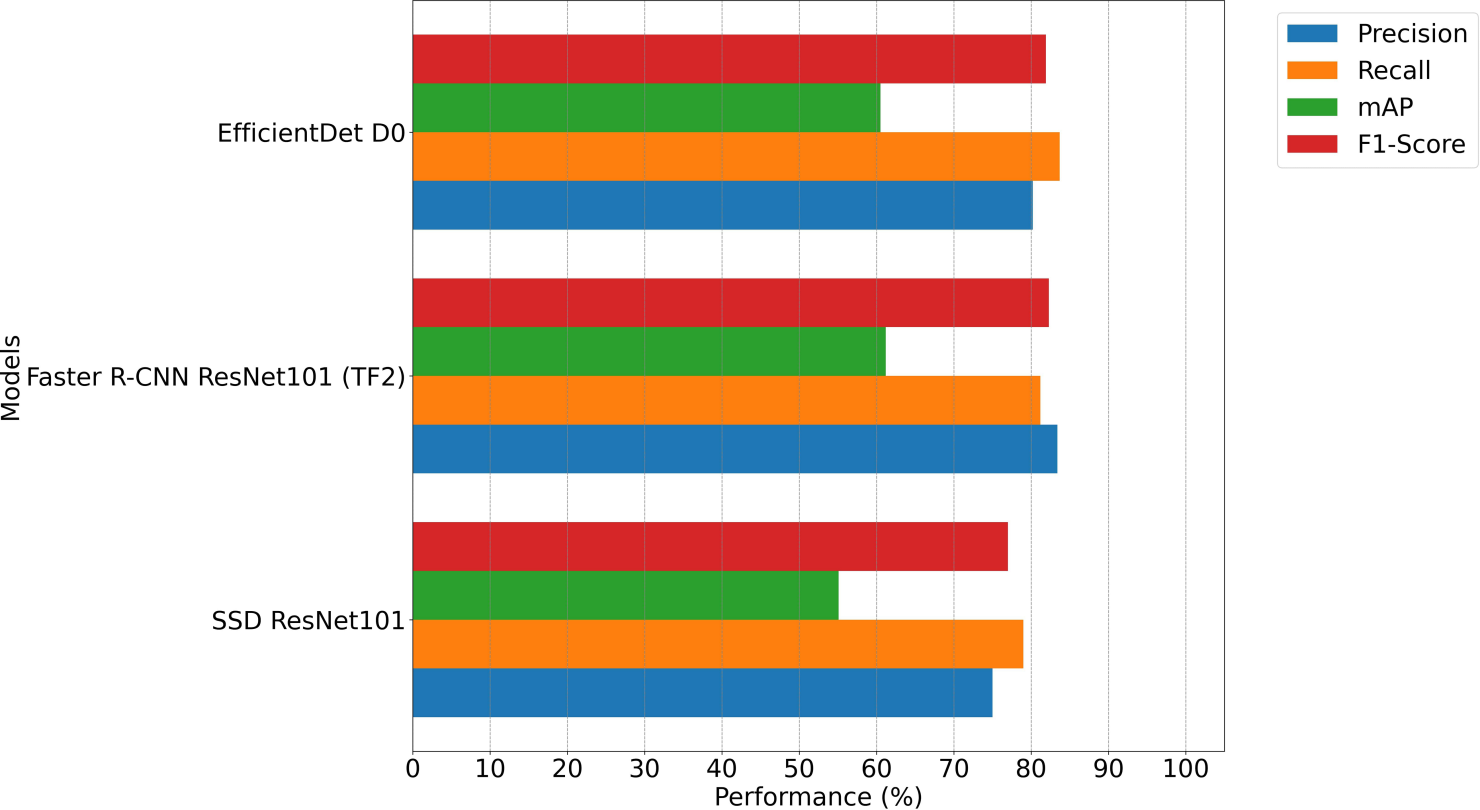
Performance comparison on Gallmann Dataset validation data.

This section presents the key findings of the model comparison. The performance of the three deep learning models is evaluated on both presented datasets in Section 2.4 (Datasets). This study focuses on the same evaluation metrics that are originally implemented in the Gallmann Phenotator Toolbox. These are precision, recall, mAP and F1-Score. Since the model decision in the Gallmann Phenotator Toolbox can be based on either the mAP or the F1-Score and since the application of this study is concerned with minimizing both false-positive and false-negative predictions, the F1-Score provides a reliable metric for evaluating the overall performance of the models. As described in Section 2.6 (Model Training), training, validation and test data were extracted from the Gallmann Dataset. In the following, results of the models on validation and test data are presented. During the training process, we monitored the validation data to identify the optimal model on the basis of the highest F1-Score for a given number of training steps. **Table 2** presents the model that achieved the highest F1-Score on validation data, along with the corresponding number of training steps in which this performance was achieved. **Figure 3** visualizes those resultson validation data. The obtained model is later evaluated on the test datasets. For validation data, Faster R-CNN demonstrated the best overall performance, achieving the highest precision (83.4%), F1-Score (82.3%), and mAP (61.2%). EfficientDet, which required the most training steps, achieved the highest recall at 83.7%, reflecting robust detection capabilities, though with a slight trade-off in precision. The SSD model, which was trained for the fewest steps, exhibited the lowest performance across all metrics.

**Table 2:** Model performances on Gallmann Dataset validation data.

Model	Steps	Precision (%)	Recall (%)	mAP (%)	F1-Score (%)
EfficientDet D0	242.500	80.2	83.7	60.5	81.9
Faster R-CNN ResNet101	130.000	83.4	81.2	61.2	82.3
SSD ResNet101	92.500	75.0	79.0	55.1	77.0

**Figure 4:**
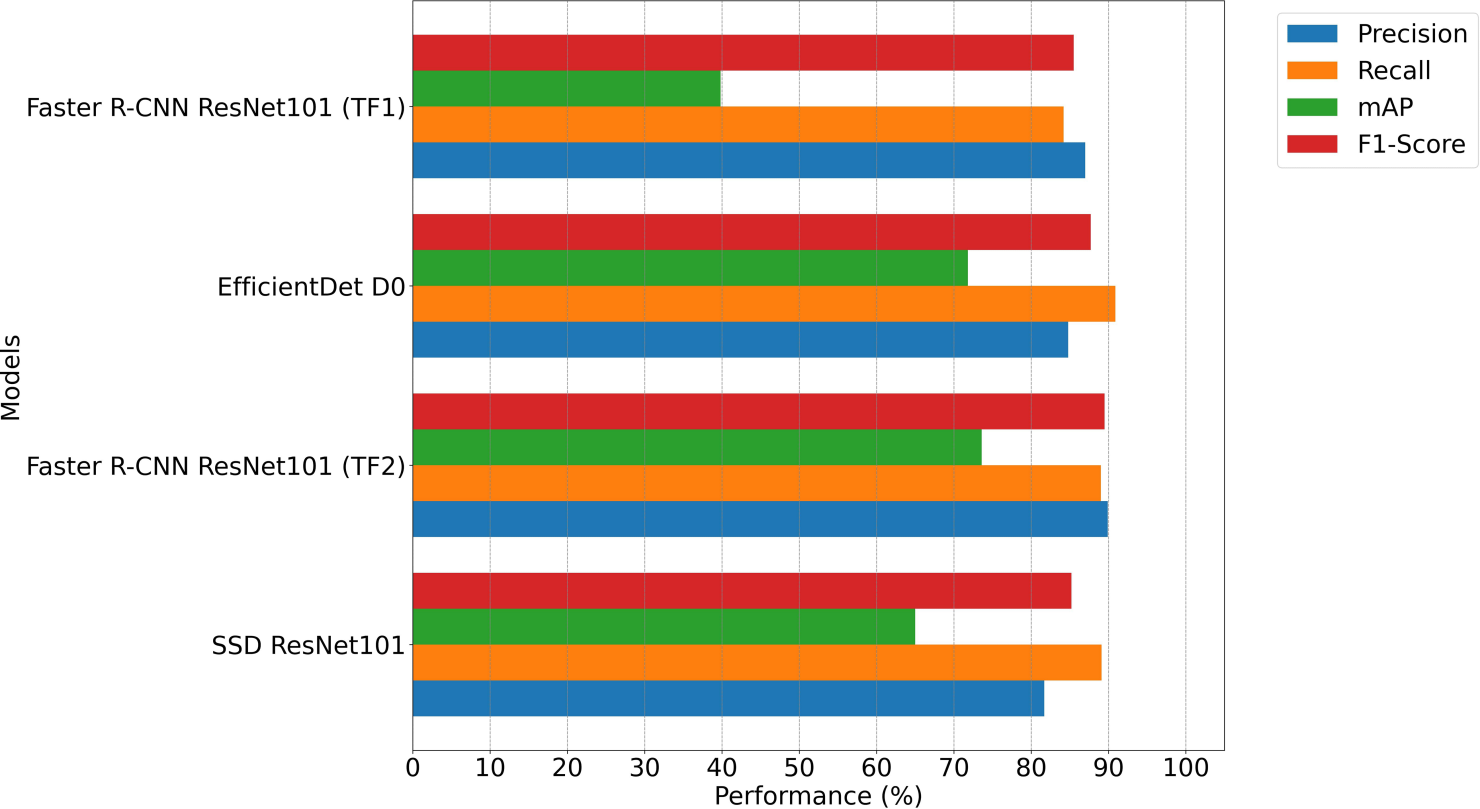
Performance comparison on Gallmann Dataset test data.

All models performed better on test data than on validation data of the Gallmann Dataset. The test data results (see **Table 3**; **Figure 4**) align with the trends observed in the validation phase. Faster R-CNN outperformed the other models, achieving a precision of 89.9%, a mAP of 73.6% and a F1-Score of 89.5%. EfficientDet also performed well. Again, it had the highest recall with 90.9%. The SSD model showed lower precision (81.7%) but maintained a high recall of 89.1%. The Faster R-CNN results on the Gallmann Dataset test data outperform those reported by Gallmann et al. (2022), who achieved an overall precision of 87%, a recall of 84.2%, a mAP of 39.8% and a F1-Score of 85.5% on test data. This improvement reflects the updates and optimizations made in the training process, highlighting the effectiveness of the Toolchain update. **Table 4** shows the confusion matrix for the best model, Faster R-CNN, on test data from the Gallmann Dataset. The matrix provides an overview of the model’s performance in correctly classifying each flower species. It also highlights potential misclassifications.

**Table 3:** Model performances on Gallmann Dataset test data.

Model	Precision (%)	Recall (%)	mAP (%)	F1-Score (%)
Faster R-CNN ResNet 101 (TF1)	87.0	84.2	39.8	85.5
EfficientDet D0	84.8	90.9	71.8	87.7
Faster R-CNN ResNet101	89.9	89.0	73.6	89.5
SSD ResNet101	81.7	89.1	65.0	85.2

**Table 4:** Confusion matrix on Gallmann Dataset test ata dwith faster R-CNN.

Prediction																
Ground Truth	Anthyllis vulneraria	Centaurea jacea	Crepis biennis	Dianthus carthusianorum	Galium mollugo	Knautia arvensis	Leucanthemum vulgare	Lotus corniculatus	Onobrychis viciifolia	Prunella vulgaris	Ranunculus	Rhinanthus alectorolophus	Salvia pratensis	Trifolium pratense	Background	False Negatives
Anthyllis vulneraria	–	–	–	–	–	–	–	–	–	–	–	–	–	–	–	–
Centaurea jacea	–	234	–	–	–	–	–	–	–	1	–	–	–	–	27	28
Crepis biennis	–	–	15	–	–	–	–	4	–	–	–	–	–	–	5	9
Dianthus carthusianorum	–	1	–	13	–	–	–	–	–	–	–	–	–	–	–	1
Galium mollugo	–	–	–	–	92	–	–	–	–	–	–	–	–	–	17	17
Knautia arvensis	–	1	–	–	–	165	–	–	–	–	–	–	–	1	8	10
Leucanthemum vulgare	–	–	–	–	–	–	539	–	–	–	–	–	1	–	35	36
Lotus corniculatus	–	–	1	–	–	–	–	569	–	–	6	–	–	–	81	88
Onobrychis viciifolia	–	–	–	–	–	–	–	–	10	–	–	–	–	–	3	3
Prunella vulgaris	–	1	–	–	–	–	–	–	–	7	–	–	–	–	1	2
Ranunculus	–	–	–	–	–	–	–	10	–	–	159	–	–	–	9	19
Rhinanthus alectorolophus	–	–	–	–	–	–	–	–	–	–	–	–	–	–	–	–
Salvia pratensis	–	1	–	–	–	–	–	–	–	–	–	–	38	–	5	6
Trifolium pratense	–	2	–	–	–	–	–	–	2	–	–	–	–	8	5	9
Background	–	20	2	4	19	7	20	90	5	2	3	–	2	2	–	–
False Positives	–	26	3	4	19	7	20	104	7	3	9	–	3	3	–	–

In addition to the evaluation on the Gallmann Dataset, the three models were tested on the Hohenheim Dataset. The model predictions on the drone images were compared with the manually counted flower data collected in the field. In this case, a correct prediction was not based on the overlap of bounding boxes but on whether the detected flowers occurred in the test quadrants where the flower data was collected. **Table 5** shows the class performance of the three models on the three flower classes - *Centaurea jacea Galium mollugo* and *Lotus corniculatus* - that were present in both the training and Hohenheim Dataset (see **Figure 1**). Notably, *Centaurea jacea*, which stands out visually, achieved high recall values across all models, with SSD reaching 100% recall. However, the precision was lower, especially for SSD (50%), indicating a high number of false positives. This suggests the models frequently misclassified other, unseen species. Often, the model predicted those unseen flowers as background. In some cases, unseen flower classes were classified as visually similar flower classes from training data. For example *Geranium pratense* was classified as *Centaurea jacea*. Another example is the unseen flower class *Galium palustre* which was often classified as *Knautia arvensis*. This suggests that the models largely rely on visual similarities between species, supporting the approach of Gallmann et al. (2022) of grouping visually similar flowers into broader classes (see **Table 1**). For *Galium mollugo*, all models performed poorly, particularly in terms of recall. Although Faster R-CNN achieved perfect precision (100%), its recall was extremely low (0.7%), leading to an F1-Score of only 1.4%. This indicates that the model only identified a very small number of actual *Galium mollugo* instances, even though when it did make a prediction, it was correct. This is also due to the fact that *Galium mollugo* was the most represented class in the manually counted data. The other models showed a similar trend, with EfficientDet and SSD also having high precision (93.8% and 92.6%, respectively) but very low recall values (1.5% and 3%), highlighting the challenge of detecting *Galium mollugo* in new environments, where variations in visual features or environmental conditions may have impacted the model’s performance. The reasons for these results will be further discussed in Section 4 (Discussion). In the case of *Lotus corniculatus*, the performance was better compared to *Galium mollugo*, but the recall remained relatively low across all models. SSD performed best, achieving the highest F1-Score (42.7%) with a recall of 27.6% and precision of 94.7%. While the models could more reliably identify *Lotus corniculatus* compared to *Galium mollugo*, the lower recall suggests that a significant number of flowers were still missed during detection, pointing to potential limitations in generalization across environments.

**Table 5:** Model performances on Hohenheim Dataset.

Model	Centaurea jacea			Galium mollugo			Lotus corniculatus		
	Precision (%)	Recall (%)	F1-Score (%)	Precision (%)	Recall (%)	F1-Score (%)	Precision (%)	Recall (%)	F1-Score (%)
EfficientDet DO	60.3	89.7	72.2	93.8	1.5	0.3	93.9	23.5	37.6
Faster R-CNN ResNet101	57.1	92.3	70.6	100	0.7	1.4	86.7	26.5	40.6
SSD ResNet101	50.0	100.0	66.7	92.6	3.0	5.8	94.7	27.6	42.7

**Table 6** shows a comparison of the model complexities in terms of the required resources. For this purpose, the storage requirements and Floating Point Operations per Second (FLOPs) of the different models were calculated. The FLOPs were calculated using the TensorFlow Profiling API. EfficientDet is the least complex in terms of both model size and FLOPs, while SSD is the most complex. These results are consistent with Tan and Le (2019), who demonstrated that EfficientDet achieves superior computational efficiency, with models requiring significantly fewer floating-point operations compared to previous object detection architectures. These differences illustrate the efficiency of the models in terms of memory and computation, which is important when choosing for specific applications.

**Table 6:** Comparison of model complexity.

Model	Model Size (MB)	FLOPs (billion)
EfficientDet	34.8	18.81
Faster R-CNN	189.0	469.71
SSD	205.0	578.27

## 4 Discussion

With these results in hand, the focus shifts to a deeper analysis of their implications and the comparative strengths and weaknesses of the different models. The comparison of Faster R-CNN, SSD, and EfficientDet highlights how their architectural model differences influence detection performance, particularly in the context of flower detection. Faster R-CNN, with its two-stage architecture, excels in precision and F1-Score on the Gallmann Dataset, making it the most accurate model for detecting flower structures. This step-by-step process allows for more accurate localization, especially in complex environments with dense vegetation. However, it is still limited in its use in real-time fieldwork (Ren et al., 2016). EfficientDet, on the other hand, based on the BiFPN architecture, strikes a balance between model complexity and performance on test data. Its weighted feature pyramid network optimizes both processing power and detection accuracy (Tan et al., 2020). Compared to SSD, EfficientDet provides more efficient feature extraction, resulting in higher recall rates without compromising precision. This makes it ideal for applications where efficient and accurate detection is critical. The extension of the Gallmann Phenotator Toolbox to support additional models increases its compatibility and flexibility of use. In addition to the models from the comparison, the TF-based code provides a solid foundation for integrating additional models from the model detection zoo, such as Mask R-CNN and CenterNet. Future work could explore model optimization, including hyperparameter tuning (Bergstra et al., 2013; Yang and Shami, 2020) or the use of different backbones, to fur- ther improve detection results. Another important direction for development involves models specifically optimized for real-time analysis, which will become increasingly relevant in agricultural applications (Chen et al., 2020). In order to efficiently apply deep learning to real-time recognition, model complexity must be reduced. A simplified model architecture enables faster processing times and better scalability (Li et al., 2022). Models such as EfficientDet from the comparative analysis are, as mentioned above, particularly well suited for this. Another promising model for real-time detection is You Only Look Once (YOLO) (Redmon et al., 2016), which despite its speed has historically struggled to accurately detect small objects due to its grid-based architecture. Recent developments, such as the optimized YOLOv3 by Liu et al. (2020), specifically address this problem and improve the detection of small targets, such as flowers in UAV images, by refining the model architecture and the feature extraction processes.

The toolchain update has improved the applicability of the Gallmann Phenotator Toolbox by ensuring compatibility with recent software. Maintaining the long-term performance and compatibility of the Phenotator Toolbox TF2 will require continuous software updates. According to Lehman (1996), software systems that interact with real-world environments must continually adapt to changing requirements to remain effective. Without regular maintenance and updates, the software’s performance will degrade as its environment changes. For scientific advancement, updating the Gallmann Phenotator Toolbox was critical (Howison et al., 2015), as it depends on components such as the Object Detection API and third-party libraries, which regularly release new versions with bug fixes and performance enhancements (Kula et al., 2018). This makes timely software updates essential. The presented practical guidelines ensure that the software is seamlessly integrated into a workflow, reducing complexity, especially in the interaction between biologists as end users and the software used (Howison et al., 2015). Although current software updates are a short-term solution, the Phenotator Toolbox TF2 will benefit from TF’s planned future developments, which promise backward compatibility and optimizations in performance and scalability^29^.

**Figure 5:**
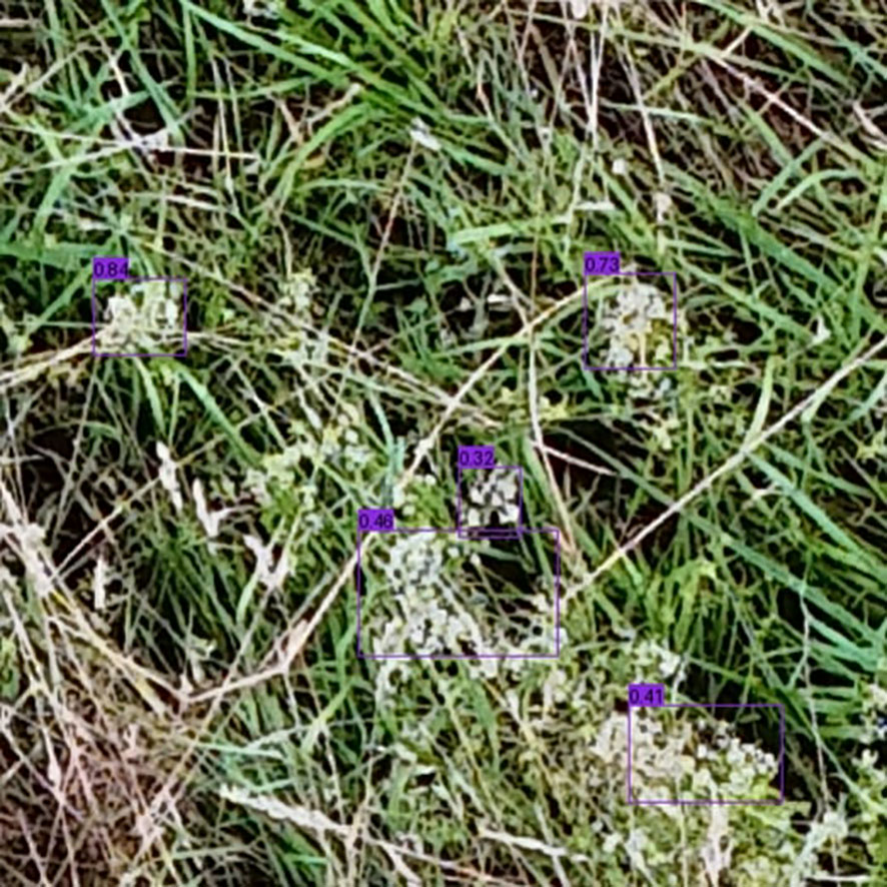
Galium Mollugo inflorescences: Prediction example of inflorescences of *Galium Mollugo* in the Hohenheim Dataset, illustrating the varying number of blossoms within each inflorescence.

While these technical adjustments have improved applicability and model performance, environmental factors remain critical challenges. The perspective of the drone is a significant limitation, especially when trying to see hidden flowers. Although drones such as the DJI Air 3 can capture high-resolution images, the view of flowers is often blocked by dense vegetation or tall grass. This occlusion makes detection more difficult, as drone imagery has a limited lateral view and cannot capture flowers that are obscured by grass. In the test environment of this study and already mentioned by Gallmann et al. (2022), this resulted in many flowers not being detected in dense grasslands, leading to misclassifications. This observation is consistent with previous studies showing that small, dense, and overlapping objects are difficult to detect in UAV imagery (Zhang et al., 2019). To address this issue, Tian et al. (2021) extended SSD based models to include a second detection step that examines potentially missed areas and specifically identifies hard-to-see objects. Although this approach has not been specifically tested on flowers, it may be a promising extension of the models to further improve the detection of flowers obscured by vegetation in dense grasslands. In the Hohenheim Dataset, the grass was dry and therefore less intensely green than in the training dataset, which can lead the model to incorrectly classify the background as a flower. Also external environmental conditions can affect detection performance. Weather conditions like rain, noise, or image blur can degrade the quality of UAV captured images (Munir et al., 2024), while different lighting conditions can impact detection performance when detecting flowers (Lin et al., 2022). A notable finding in the results was the poorer class recognition in the Hohenheim Dataset, which was constrained by the availability of annotated training data (Gallmann et al., 2022). This limits the model’s ability to detect flower species that were not part of the training set. Expanding the dataset to include a wider variety of flower species is necessary to improve the generalizability of the model. The Hohenheim Dataset contains several flower species that were not present in the Gallmann Dataset training data. Including these new classes in future training sessions would allow the model to recognize a broader range of species, thus improving accuracy and extending its applicability to diverse ecological settings. In addition to new flower classes, future training datasets should include flowers in different growth stages and under different seasonal and climatic conditions (Katal et al., 2022), since the Hohenheim Dataset includes vegetation in dry periods. Currently, the approach focuses on detecting and classifying different classes of flowers. However, for applications where only the total number of flowers is of interest, it may be more efficient to consider binary classification - distinguishing between ‘flower’ and ‘non-flower’ – to simplify the task. Similar to the work of Ayhan et al. (2020), who effectively used binary classification for vegetation detection, this approach could reduce model complexity and enhance efficiency by focusing solely on flower presence. The manually collected data showed that some test plots contained *Galium Mollugo* flowers that were not in bloom at the time of capture, making recognition by deep learning models difficult. These models were mainly trained on plants in full bloom recorded between May 23 and July 3 (Gallmann et al., 2022), which affects their performance in detecting plants at other stages of development, such as in the Hohenheim Dataset recorded in mid-July. As outlined in Section 2.1 (Literature Review) features play a key role in object detection. If these characteristics, such as fully opened flower, are not present, it is difficult for the model to correctly recognize the object. Another factor contributing to the poor recognition of *Galium Mollugo* is that the models were trained to recognize inflorescences rather than individual flowers (Gallmann et al., 2022). In contrast, the manual data collection for the ground truth in the Hohenheim Dataset involved counting individual flowers instead of inflorescences. Therefore, this mismatch between what the model was trained to detect and what was counted during ground truth data collection introduces a significant source of error. When the model attempts to detect entire inflorescences (see **Figure 5**), but the manual annotations reflect individual flowers, the predictions are misaligned with the ground truth.

Expanding and adapting training data would also enable the Phenotator Toolbox TF2 to be applied to more specific scenarios, such as the detection of invasive species (De Sá et al., 2018; Bakacsy et al., 2023; Sarkar and Kelley, 2023). Additionally, incorporating multisensor data, such as hyperspectral images, alongside standard RGB data, could further enhance the system’s capabilities. Hyperspectral data, for example, have shown promising results in flower recognition (Landmann et al., 2015; Yao et al., 2023) by providing a richer environmental context, potentially improving detection accuracy in complex ecosystems. Beyond dataset expansion, transfer learning is increasingly applied in remote sensing and could therefore improve the applicability of the Phenotator Toolbox TF2 to different environmental contexts. It reduces the need to generate large amounts of newly labeled data, which is often a challenge in large scale applications. Model fine-tuning is the most commonly used transfer learning application in biodiversity assessment. Its success is limited by reduced real-time performance. Unsupervised Domain Adaptation, another transfer learning approach, provides the ability to adapt images to different weather or lighting conditions (Ma et al., 2024).

## 5 Conclusion

In summary, this work successfully updated and extended the Gallmann Phenotator Toolbox to the Phenotator Toolbox TF2 by integrating up-to-date software and different models, improving applicability and flexibility in application. The migration to TF 2 enabled the use of recent software packages and significantly improved model training, enhancing both usability and performance. The planned updates of TF regarding backward compatibility are promising for the continued use of Phenotator Toolbox TF2. The comparative analysis showed that Faster R-CNN, with its high precision, was the most reliable model for flower detection in grasslands. EfficientDet had the best recall, making it ideal for maximizing detection rates. Its reduced complexity also enhances its suitability for efficient flower detection tasks. However, SSD lagged behind in both precision and recall, indicating that it is less suitable for environments where detection accuracy is critical. Despite the technical advances, several challenges remain. Detection performance was lower in dense vegetation and among non-flowering plants, suggesting that improvements in occlusion handling and inclusion of more seasonal and climatic as well as flower class variations in the dataset are necessary. Furthermore, incorporating more flower species into the training data will help generalize the model’s applicability to diverse ecological settings. The provided guidelines offer actionable steps for biologists and ecologists, bridging the gap between machine learning techniques and real-world conservation efforts. The introduction of new flower classes and different environmental conditions of the Hohenheim Dataset highlighted the need for further refinement. These results underscore the potential of integrating UAVs and machine learning to transform large-scale biodiversity monitoring, offering a scalable solution to the urgent challenge of pollinator decline.

## Data Availability

The dataset from “Flower Mapping in Grasslands With Drones and Deep Learning” (Gallmann et al., 2022) must be requested from the respective authors, while our dataset is available upon request. Requests to access the datasets should be directed to MS, schnalke.marie@web.de. The code supporting this study, as well as the detailed setup instructions, is publicly available at: https://github.com/marieschnalke/Phenotator-Toolbox-TF2. For further inquiries or access, please contact the corresponding author.

## Footnotes

1 The BeeVision Project is funded by the Carl-Zeiss-Stiftung (more information at: https://www.carl-zeiss-stiftung.de/themen-projekte/uebersicht-projekte/detail/beevision-monitoring-der-bestaeuber-vielfalt-durch-ynamic-vision, accessed on 02.09.2024).

2 More information at: https://github.com/gallmann/Phenotator-Toolbox , accessed on 11.06.2024.

3 More information at: https://github.com/tensorflow/models/blob/master/research/object_detection/g3doc/tf1_detection_zoo.md , accessed on 15.08.2024.

4 An Open-Source-Platform for machine learning (More information at: https://www.tensorflow.org/, accessed on 16.09.2024).

5 More information at: https://github.com/tensorflow/models/tree/master/research/object_detection, accessed on 02.09.2024.

6 More information at: https://www.tensorflow.org/guide/migrate, accessed on 30.08.2024.

7 Local shape/texture, edge shape, spatial distribution of flowers, and color.

8 More information at: https://www.dji.com/de/air-3/specs, accessed on 24.01.2025.

9 More information at: https://eur-lex.europa.eu/eli/reg_impl/2019/947/oj, accessed on 16.09.2024.

10 More information at: https://www.tensorflow.org/install/source#gpu, accessed on 30.08.2024.

11 More information at: https://www.tensorflow.org/guide/gpu, accessed on 10.09.2024.

12 More information at: https://blog.tensorflow.org/2019/09/tensorflow-20-is-now-available.html, accessed on 02.09.2024.

13 More information at: https://www.tensorflow.org/guide/intro_to_graphs, accessed on 16.09.2024.

14 More information at: https://www.tensorflow.org/guide/keras, accessed on 02.09.2024.

15 More information at: https://github.com/tensorflow/models/blob/master/research/object_detection/g3doc/tf2_detection_zoo.md, accessed on 15.08.2024.

16 More information at: https://www.tensorflow.org/guide/data, accessed on 10.09.2024.

17 More information at: https://www.tensorflow.org/guide/distributed_training , accessed on 10.09.2024.

18 More information at: https://www.tensorflow.org/guide/migrate, accessed on 30.08.2024.

19 More information at: https://github.com/marieschnalke/Phenotator-Toolbox-TF2 .

21 More information at: https://github.com/OpenDroneMap/WebODM, accessed on 04.09.2024.

22 A step refers to the processing of a batch of images, with the model updating its parameters after each step (Abadi et al., 2016).

23 More information at: https://github.com/tensorflow/models/blob/master/research/object_detection/g3doc/tf2_detection_zoo.md, accessed on 15.09.2024.

24 The output of a convolutional layer in a CNN, highlighting key features in the input image (O’Shea and Nash, 2015).

25 More information at: https://www.gesetze-im-internet.de/luftvo_2015/ accessed on 14.09.2024.

26 More information at: https://www.gesetze-im-internet.de/luftvg/, accessed on 14.09.2024.

27 More information at: https://www.dipul.de/homepage/de/, accessed on 04.09.2024.

28 More information at: https://www.dronedeploy.com/, accessed on 10.09.2024.

29 More information at: https://blog.tensorflow.org/2022/10/building-the-future-of-tensorflow.html , accessed on 06.09.2024.
